# Transcriptomic Analysis Reveals the Role of *TRIM26* in Hepatocellular Carcinoma and Its Association With the Wnt/*β*‐catenin Signaling Pathway

**DOI:** 10.1155/humu/3090777

**Published:** 2026-04-24

**Authors:** Conghua Song, Zijun Hou, Huifeng Wu, Xiaomei Li

**Affiliations:** ^1^ Gastrointestinal Endoscopy Center, The Affiliated Hospital of Putian University, Putian City, Fujian Province, China, ptu.edu.cn; ^2^ Key Laboratory of Translational Tumor Medicine in Fujian Province, Putian University, Putian City, Fujian Province, China, ptu.edu.cn; ^3^ College of Environmental and Biological Engineering, Putian University, Putian City, Fujian Province, China, ptu.edu.cn; ^4^ School of Basic Medicine, Putian University, Putian City, Fujian Province, China, ptu.edu.cn

**Keywords:** HCC, immune infiltration, single-cell analysis, *TRIM26*, Wnt/*β*-catenin signaling pathway

## Abstract

**Background:**

Hepatocellular carcinoma (HCC) shows high incidence and mortality worldwide. *TRIM26*, an E3 ubiquitin ligase within the TRIM family, exerts regulatory functions in various tumors. This study analyzed the expression patterns and potential functions of *TRIM26* in HCC based on transcriptomic data.

**Methods:**

First, the differential expression of TRIM26 between tumor and normal tissues was analyzed using the TCGA dataset and cross‐validated using TIMER 2.0 and HCCDB. Enrichment analysis evaluated its association with hallmark pathways including Wnt/*β*‐catenin. A gene functional interaction network was built via GeneMANIA to explore TRIM26 and the Wnt/*β*‐catenin pathway. Immune cell infiltration was quantified by ssGSEA for immune microenvironment correlation. scRNA‐seq data established an HCC single‐cell atlas to define TRIM26 distribution across cell subsets. AUCell was used to assess TRIM26‐pathway associations within specific cell types.

**Results:**

TRIM26 was significantly upregulated in HCC tissues, and its high expression correlated with enrichment of oncogenic pathways including Wnt/*β*‐catenin, G2/M checkpoint, and TGF‐*β*. GeneMANIA showed that TRIM26 interacted directly or indirectly with Wnt/*β*‐catenin core molecules, implying its regulatory role. TRIM26 expression was closely linked to infiltration of activated B cells, CD8^+^ T cells, and NKT cells. Single‐cell analysis revealed TRIM26 was mainly expressed in hepatocytes, T/NK cells, myeloid cells, and B cells. Importantly, in hepatocytes, TRIM26 strongly correlated with Wnt/*β*‐catenin activity, which was much higher in tumor hepatocytes than normal ones.

**Conclusion:**

In HCC, TRIM26 was abnormally overexpressed. TRIM26 may regulate tumor progression via the Wnt/*β*‐catenin pathway and is linked to immune infiltration. Thus, TRIM26 is a potential therapeutic target for HCC.

## 1. Introduction

Globally, an estimated 906,000 new cases of HCC and 830,000 related deaths were recorded in 2020 [1]. As the sixth most frequently diagnosed cancer [2] and the third leading cause of global cancer‐associated mortality, HCC has shown a rapidly increasing incidence in recent years [3]. Due to the absence or atypia of symptoms, the diagnosis of HCC is often challenging [4]. Early diagnosis of HCC remains challenging, largely due to the absence or atypical presentation of initial symptoms. Common early manifestations include abdominal pain, weight loss, and fatigue, whereas advanced disease may present with jaundice, ascites, and fever [5, 6]. At present, surgery remains the primary treatment method for achieving long‐term survival of HCC patients; however, the 5‐year postoperative survival rate of HCC ranges from 20% to 60% [7–9]. A lack of typical manifestations in early‐stage HCC often leads to a late diagnosis, with only 30%–40% of patients considered eligible for surgical treatment [10, 11].

As a member of the TRIM family, *TRIM26* shares similar structural characteristics with other TRIM proteins [12, 13]. Previous studies have found that *TRIM26* promotes the ubiquitination of *β*‐catenin, thereby suppressing the progression of HCC [14]. Functioning as an E3 ligase, *TRIM26* exerts inhibitory effects on liver cancer by facilitating the ubiquitination and degradation of ZEB1. It has also been reported to co‐regulate ZEB1 expression in coordination with the deubiquitinase USP39, thereby influencing the progression of HCC [15]. In various cancers, *TRIM26* overexpression can inhibit the proliferation of various types of cancer cells [15–17]. The Wnt/*β*‐catenin signaling pathway is a key driver of HCC initiation and progression [18]. However, whether *TRIM26* participates in regulating this pathway in a broader manner beyond its direct control of *β*‐catenin, as well as its potential role in shaping the tumor immune microenvironment, remains largely unexplored. Therefore, a comprehensive investigation into the expression pattern of *TRIM26* in HCC and its association with the Wnt/*β*‐catenin signaling pathway is crucial for elucidating its potential value as a therapeutic target.

Therefore, the purpose of this study was to explore the expression pattern of *TRIM26* in HCC and its association with tumor biological behavior. Multiple public databases were utilized to develop a single‐cell atlas of HCC, enabling systematic evaluation of the correlations between *TRIM26* expression and key features including immune infiltration and Wnt/*β*‐catenin signaling pathway activity. This framework is expected to deepen the understanding of the immunobiology of HCC, providing novel insights for the development of precise prognostic tools and therapeutic strategies in HCC treatment.

## 2. Materials and Methods

### 2.1. Data Acquisition

The RNA‐seq data corresponding to TCGA‐liver hepatocellular carcinoma (LIHC) were acquired through the application programming interface (API) of the TCGA GDC. After eliminating samples lacking clinical follow‐up information, those with a survival of > 0 days were kept. Ensembl identifiers were converted into gene symbols. For genes matched to multiple gene symbols, the maximum expression value was retained. After initial filtering, 365 primary tumor samples were included in the study.

The scRNA‐seq data of eight primary liver cancer samples and eight matched adjacent nontumor liver samples in GSE149614 were retrieved from the Gene Expression Omnibus (GEO) database [19]. The expression data of *TRIM26* were extracted from the TIMER2.0 database [20] (http://timer.cistrome.org/).

### 2.2. Filtering, Dimensionality Reduction, and Clustering

The scRNA‐seq data from GEO were preprocessed. The data were read via the Read10X function in the Seurat package [21]. Quality control filtering was performed according to the following criteria: genes detected in fewer than three cells were excluded, and cells were retained if they expressed between 200 and 10,000 genes and had a mitochondrial gene proportion of < 15%. Data normalization was conducted with the SCTransform function. Subsequently, principal component analysis (PCA) was performed using the RunPCA function, followed by batch effect removal employing the harmony package [22]. The Top 30 PCs were subsequently used for UMAP dimensionality reduction. Cell clusters were identified via the FindNeighbors and FindClusters functions (at resolution of 0.1). Finally, cell types were annotated by referencing canonical marker genes from the CellMarker2.0 database [23]. Quality control indicators for different cell subpopulations are shown in Figure S1.

### 2.3. Pathway Analysis and Differential Gene Enrichment Analysis

HALLMARK pathway scores was calculated using the GSVA package [24], with gene sets sourced from the MSigDB. The TCGA cancer samples were assigned based on the median *TRIM26* expression into low‐ and high‐expression groups. Differential analysis was conducted utilizing the limma package [25] under the criteria of FDR of < 0.05 and |log2 *F* *C*| > log2 (1.5). GSEA was subsequently conducted with the clusterProfiler package [24].

### 2.4. Immune Infiltration Analysis

The enrichment scores of immune cells in each sample were calculated using ssGSEA, with immune cell gene derived from the reference literature [26].

### 2.5. Statistical Analysis

Statistical analysis was conducted in R language. Intergroup comparisons of continuous variables were performed using the Wilcoxon rank‐sum test, and correlations were assessed using Spearman′s method. *p* < 0.05 signified statistical significance.

## 3. Results

### 3.1. Expression Characteristics of *TRIM26* in Pan‐Cancer and HCC and Its Clinical Correlation

**Figure 1:**
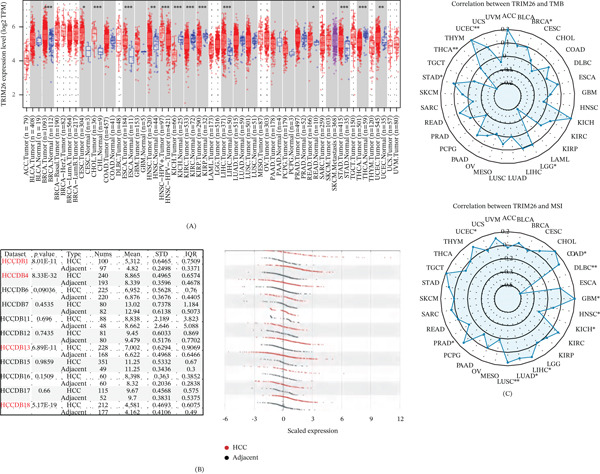
*TRIM26* expression in pan‐cancer and liver hepatocellular carcinoma. (A–B) *TRIM26* expression in pan‐cancer. (C) Radar chart showing the association between TMB, MSI, and *TRIM26* expression.

*TRIM26* expression data were acquired from the TIMER2.0 database. Elevated expression of *TRIM26* was observed in multiple tumor types in comparison with the normal group, including LIHC, BRCA, CESC, CHOL, ESCA, and STAD (Figure 1A). Consistently, analysis based on the HCCDB database revealed pronounced upregulation of *TRIM26* in HCC than in the adjacent normal group across several datasets (HCCDB1, HCCDB4, HCCDB13, and HCCDB18) (Figure 1B). Furthermore, a positive correlation between *TRIM26* expression and microsatellite instability (MSI) in LIHC, LUSC, LUAD, and UCEC was observed (Figure 1C).

### 3.2. Enrichment Analysis of *TRIM26* Expression and Wnt/*β*‐catenin Signaling Pathway Activity

**Figure 2:**
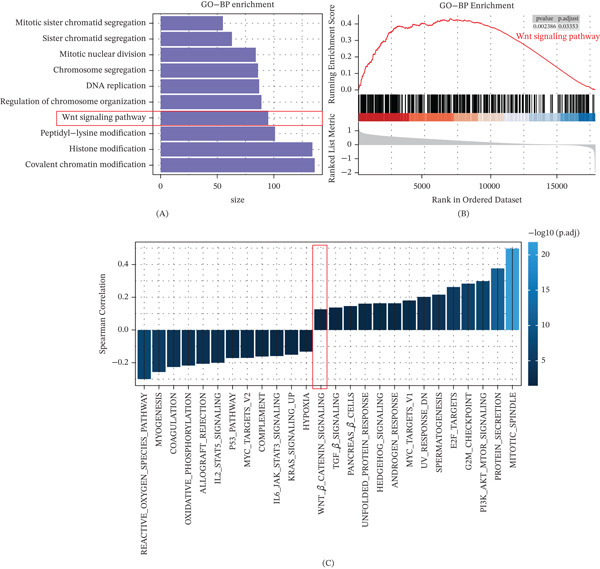
GSEA and pathway correlation analysis of *TRIM26* high‐ and low‐expression groups. (A–B) Enrichment analysis of DEGs between the expression groups of *TRIM26*. (C) Correlation between *TRIM26* and hallmark pathway scores.

Based on the TCGA dataset, tumor samples were stratified based on median *TRIM26* level into high‐ and low‐expression groups. According to differential expression analysis, we identified 37 downregulated and 2239 upregulated genes. Enrichment analysis demonstrated that these DEGs were closely involved in biological processes (BPs) including Wnt signaling, histone modification, and DNA replication (Figure 2A,B). Furthermore, GSEA confirmed significant enrichment of the Wnt signaling pathway in samples with high *TRIM26* expression. Subsequent correlation analysis using Spearman′s method revealed a significant positive association between *TRIM26* expression and activity scores of both the Wnt signaling pathway and some other HALLMARK pathways (Figure 2C).

### 3.3. Association Analysis of *TRIM26* Expression and Immune Cell Infiltration in HCC

**Figure 3:**
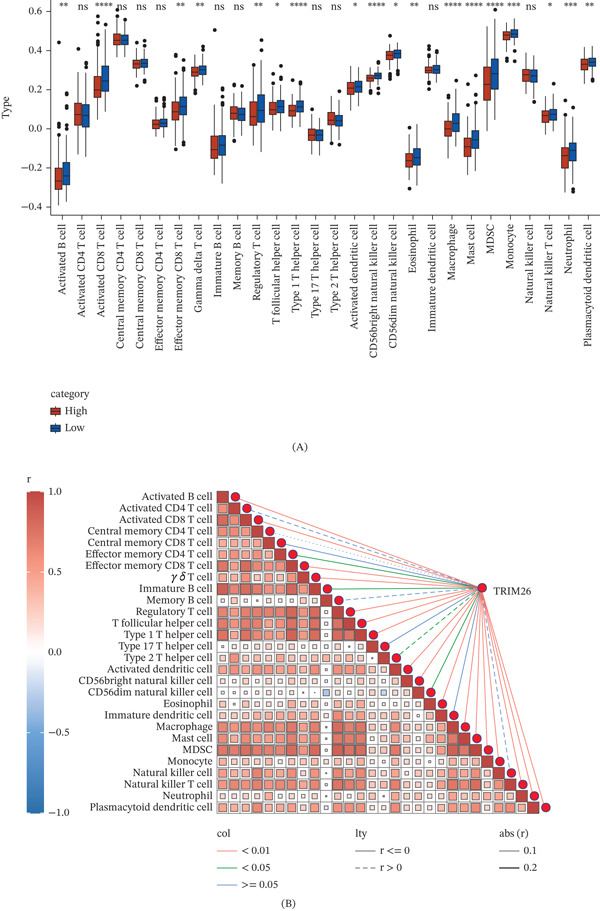
Correlation analysis of immune cell infiltration. (A) Differences in immune infiltration between the expression groups of *TRIM26*. (B) Association between *TRIM26* and immune cell scores. ∗*p* < 0.05, ∗∗*p* < 0.01, and ∗∗∗*p* < 0.001.

Immune cell infiltration in both high and low *TRIM26* expression groups was computed via ssGSEA and compared. It was observed that samples with lower *TRIM26* expression had markedly higher infiltration of activated B cells, activated CD8 T cells, regulatory T cells, mast cells, macrophages, and neutrophils (Figure 3A). Furthermore, correlation analysis between *TRIM26* expression and immune cell infiltration demonstrated that *TRIM26* expression was markedly linked to various immune cell types, including NKT cells, activated B cells, and activated CD8 T cells (Figure 3B).

### 3.4. The Association Between *TRIM26* and the Wnt/*β*‐catenin Pathway in HCC Based on GeneMANIA Analysis

**Figure 4:**
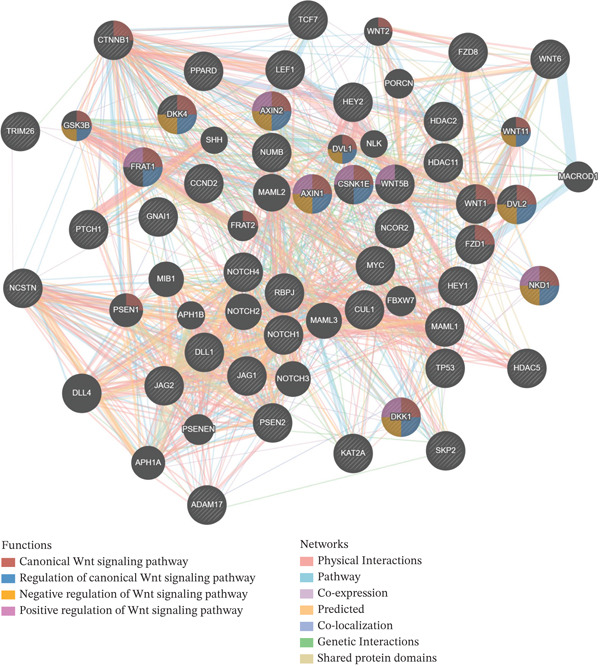
Network diagram of *TRIM26* and genes linked to Wnt/*β*‐catenin signaling pathway; lines of different colors represent different approaches to obtain correlations.

To investigate whether *TRIM26* was involved in regulating the Wnt/*β*‐catenin signaling pathway, the *TRIM26* gene and the HALLMARK_WNT_BETA_CATENIN_SIGNALING gene set were both imported into the GeneMANIA platform to construct a gene functional interaction network (Figure 4). The results showed that *TRIM26* was functionally connected with key genes (e.g. *FRAT1*, *NCSTN*, and *CTNNB1*) in the Wnt/*β*‐catenin pathway, suggesting that *TRIM26* may affect the activity of core components of this signaling pathway.

### 3.5. Construction of an HCC Atlas Based on scRNA‐Seq Data and Cell Type Distribution Characteristics of *TRIM26*

**Figure 5:**
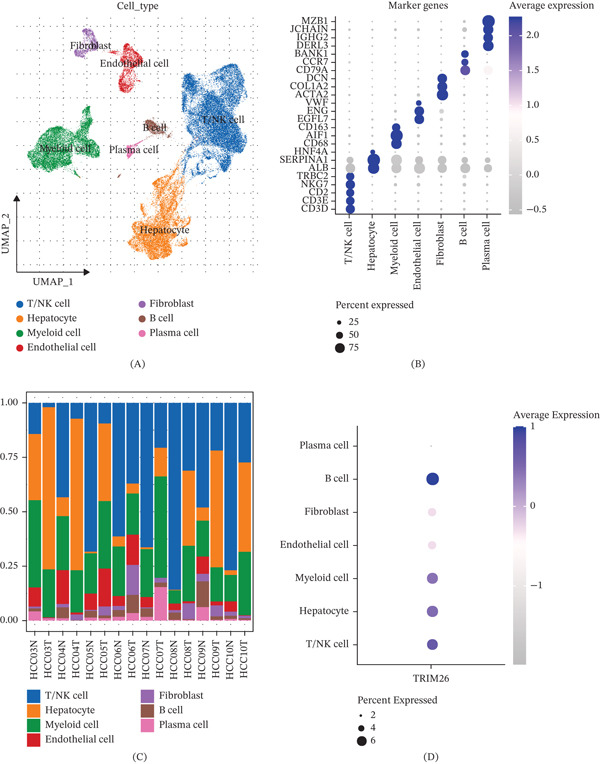
Single‐cell atlas of HCC. (A) UMAP plot of annotated cell subsets; (B) specifically high‐expressed genes in different cell types; (C) proportion of cell subsets in different samples; and (D) *TRIM26* expression in different cell clusters.

After cell filtering, data standardization, dimensionality reduction, and cluster analysis, 54,481 high‐quality cells were retained for subsequent analyses. Based on the expression of classical marker genes in each cluster, the cells were classified into seven major immune cell types, including hepatocytes, T/NK cells, myeloid cells, fibroblasts, plasma cells, endothelial cells, and B cells (Figure 5A–C). Specifically, hepatocytes were identified by the expression of *ALB*, *SERPINA1*, and *HNF4A*; myeloid cells were identified by the expression of *CD68*, *AIF1*, and *CD163*; endothelial cells were identified by the expression of *EGFL7*, *ENG*, and *VWF*; fibroblasts were identified by the expression of *ACTA2*, *COL1A2*, and *DCN*; and B cells were identified by the expression of *CD79A*, *CCR7*, and *BANK1* (Figure 5B). Analysis of the seven immune cell types showed that hepatocytes and T/NK cells accounted for a relatively higher proportion (Figure 5C). Further analysis of the expression of *TRIM26* in different cell types showed that *TRIM26* was mainly expressed in myeloid cells, B cells, hepatocytes, and T/NK cells (Figure 5D).

### 3.6. Correlation Between *TRIM26* and Hallmarks in Hepatocytes

**Figure 6:**
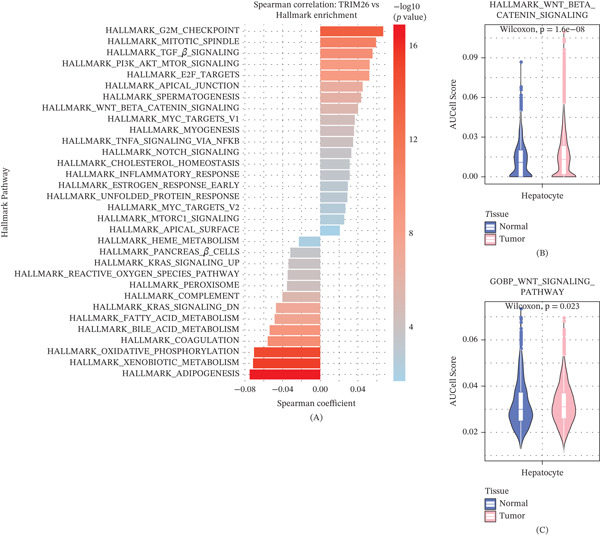
The correlation between *TRIM26* and hallmark. (A) Spearman correlation between *TRIM26* expression and hallmark enrichment scores in hepatocytes. (B–C) Expression of genes associated with WNT_BETA_CATENIN_SIGNALING in hepatocytes from normal and tumor groups.

To characterize hallmark pathway enrichment patterns across different cellular states, we performed pathway activity analysis on single‐cell hepatocyte samples using the AUCell algorithm. Following the calculation of enrichment score for all hallmark pathways, Spearman correlation between *TRIM26* expression and the enrichment scores was further calculated (Figure 6A). We found that *TRIM26* was significantly correlated with multiple cancer‐related pathways, with strong positive correlation with signaling pathways such as HALLMARK_TGF_BETA_SIGNALING, HALLMARK_WNT_BETA_CATENIN_SIGNALING, and HALLMARK_G2M_CHECKPOINT. Notably, the WNT/*β*‐catenin signaling pathway enrichment score was significantly higher in tumor‐derived hepatocytes than in normal hepatocytes (Figure 6B,C). Together with the positive correlation between *TRIM26* and pathway activity, these findings showed that *TRIM26* was possibly functionally associated with the activation of this pathway, and that the WNT/*β*‐catenin signaling pathway may be a potential mediating pathway for its involvement in tumor BPs.

## 4. Discussion

HCC is the most frequent liver cancer type worldwide [27–29]. At present, the 5‐year survival rate for HCC patients is approximately 20%, and the prognostic outcomes vary significantly across different tumor stages [30–32]. Therefore, early detection and diagnosis are crucial for improving prognosis. Here, this study systematically characterized the expression profile and biological functions of *TRIM26* in HCC. Multicenter data analysis revealed that *TRIM26* was significantly upregulated in HCC tissues. Importantly, high *TRIM26* expression was closely linked to Wnt/*β*‐catenin pathway activity and infiltration of multiple immune cell types in HCC. Single‐cell analysis further revealed that *TRIM26* was primarily expressed in hepatocytes, T/NK cells, myeloid cells, and B cells. Particularly, its expression was positively correlated with Wnt/*β*‐catenin pathway activity in hepatocytes. These findings suggest that *TRIM26* may serve as a key molecular link between the Wnt/*β*‐catenin signaling pathway and the tumor immune microenvironment. It holds promise as a potential therapeutic target for HCC and a predictive biomarker for immunotherapy response, laying a theoretical foundation for subsequent functional validation and clinical translational research.

As an E3 ubiquitin ligase within the TRIM family, *TRIM26* plays regulatory roles in various BPs, for instance, cell cycle, proliferation, DNA repair, ferroptosis, and autophagy [33]. In cancer, *TRIM26* has been described as a tumor‐suppressor in HCC, where it was found to promote ferroptosis, and its downregulation was associated with poor prognosis in HCC [34, 35]. This tumor‐suppressive role was further supported by another study demonstrating that *TRIM26* inhibited the growth and migration of HCC by ubiquitinating and degrading *ZEB1*, a key oncogenic transcription factor for HCC growth and metastasis [15, 36]. It has also been reported that *TRIM26* exhibited oncogenic effects on different tumor types. The AKT/GSK3*β*/*β*‐catenin pathway could be blocked upon *TRIM26* knockdown, thereby suppressing bladder cancer cell proliferation, migration, and invasion [17]. These aforementioned results suggest that the oncogenic or tumor‐suppressive role of *TRIM26* may be dependent on the type of tissue, cell, or tumor. Though existing studies revealed the involvement of some TRIM family proteins in tumor progression by regulating the Wnt pathway, whether *TRIM26* participates in tumor progression by regulating the Wnt pathway remains unclear.

The Wnt/*β*‐catenin signaling pathway plays a crucial role in both physiological and pathological processes [37–40]. In the liver, this pathway participates in hepatic metabolic zonation, homeostatic renewal, and postinjury regeneration [41] and regulates liver physiological functions [42]. Additionally, activation of this pathway is essential for coordinating liver regeneration, especially after partial hepatectomy or toxic injury [43]. At the pathological level, aberrant activation of the Wnt/*β*‐catenin pathway has been considered one of the major genetic alterations in human HCC as it can synergize with multiple signaling pathways to promote HCC formation and exert carcinogenic effects through downstream effectors [44]. However, though aberrant pathway activation might serve as a hallmark for various tumors and liver diseases, its functions are highly complex, context‐dependent, and tightly regulated, influencing cellular functions and intercellular adhesion [45].

Regarding its regulatory mechanisms, ubiquitination serves as a core process controlling *β*‐catenin levels within the pathway. In the absence of Wnt signaling, *β*‐catenin is sequentially phosphorylated by CK1 and GSK3, subsequently recognized by the E3 ligase *β*‐Trcp for ubiquitination, and ultimately degraded by the 26S proteasome [46]. Furthermore, multiple proteins participate in disease processes by regulating this pathway. Specifically, highly expressed USP25 has been shown to interact with *TRIM21* to activate the Wnt/*β*‐catenin pathway and downstream proteins, thereby promoting the proliferation and migration of liver cancer cells and contributing to a poor prognosis [37]. Notably, *TRIM26* has also been linked to this pathway, as its deletion can reduce *β*‐catenin ubiquitination in hepatocytes and promote hepatocyte proliferation through Wnts secreted by M1 macrophages, suggesting that loss or inhibition of *TRIM26* may contribute to liver cancer [12]. However, in our study, *TRIM26* was found to be upregulated in HCC and positively correlated with Wnt/*β*‐catenin activity, a finding that appears to contrast with its role as a tumor‐suppressor that promotes *β*‐catenin degradation. Two potential explanations may account for this discrepancy. First, the functional role of TRIM26 may be cell type–specific. For instance, *TRIM26* deficiency in macrophages has been shown to enhance M1 polarization and Wnt secretion, activating Wnt signaling in adjacent hepatocytes [12], suggesting that immune‐derived signaling could drive the observed correlation. Additionally, a feedback mechanism may exist wherein abnormal Wnt activation, which is typically driven by CTNNB1 or AXIN1 mutations [47, 48], could induce compensatory *TRIM26* upregulation. However, mutant *β*‐catenin evades degradation [12], leading to coexistence of high *TRIM26* levels and sustained pathway activity. Collectively, the function of *TRIM26* in HCC might be context‐dependent, which, however, requires further study in a cell type–specific manner.

In this study, *TRIM26* was mainly expressed in myeloid cells, B cells, hepatocytes, and T/NK cells. T cells can be divided into *αβ* and *γδ* subtypes, among which *γδ* T cells account for approximately 15%–25% of liver T cells. In liver diseases, these cells can both exert protective effects and cause detrimental impacts, showing dual immune functions [49]. NKT cells, a subset of T lymphocytes and the most abundant lymphocyte population in the liver, are characterized by a high degree of heterogeneity and coexpression of surface markers typical of both T cells and NK cells [49]. Hepatocytes are the primary cellular component of the liver, and play a key role in liver regeneration due to their distinctive proliferative ability [50]. This cell type maintains lipid homeostasis by regulating lipases, which is crucial for energy supply and metabolic substrates in liver regeneration [51, 52]. The expression of *TRIM26* is significantly correlated with various immune cell types, such as activated B cells, activated CD8^+^ T cells, and NKT cells. As antigen‐presenting cells (APCs), B cells can activate T cells and enhance cellular immune responses [53]. Activated B cells differentiate into plasma cells, which secrete antitumor antibodies to act directly on tumor cells and inhibit their growth [53]. Studies showed that in HCC, activated CD8^+^ T cells regulate immune responses through interactions with other immune cells (e.g., dendritic cells and regulatory T cells) [54]. NKT cells recognize lipid antigens presented by CD1d molecules through their T cell receptors (TCRs), thereby activating antitumor immune responses. This recognition mechanism enables NKT cells to respond rapidly and attack liver cancer cells [55]. In HCC, antitumor effects are mediated by NKT cells via direct cytotoxicity and immunomodulatory mechanisms. Studies indicated that NKT cell activity is linked to patient prognosis, with elevated infiltration levels typically associated with improved clinical outcomes [56]. The activity of some other immune cells is suppressed by regulatory T cells via direct cell contact and secretion of immunosuppressive cytokines such as TGF‐*β* and IL‐10 [57]. Mast cells regulate the TME and promote tumor progression by secreting various cytokines (e.g., histamine, IL‐4, IL‐6, and IL‐10) and chemokines (e.g., CXCL10, CCL3, and CCL5) [58].

Despite the progress made in analyzing the role of *TRIM26* in HCC and its association with the Wnt/*β*‐catenin signaling pathway, some limitations should be acknowledged. First, this study primarily analyzed transcriptome data from public databases and lacked direct validation of *TRIM26* function through in vitro and in vivo experiments. Subsequent work will employ molecular biological approaches such as gene knockdown/overexpression, Western blot analysis, and luciferase reporter assays to validate *TRIM26*′s regulatory role in Wnt/*β*‐catenin pathway activity within HCC cell lines. Secondly, although scRNA‐seq analysis revealed the expression distribution of *TRIM26* across different cell subpopulations, it failed to elucidate its specific functions in cell–cell interactions. Moving forward, we plan to integrate ligand–receptor analysis tools with coculture systems to explore the regulatory networks of *TRIM26* within the tumor immune microenvironment. Third, the public databases utilized in this study were primarily derived from Western populations or specific cohorts, potentially introducing sample selection bias. Subsequent validation should incorporate independent, multicenter clinical cohorts with large sample sizes and more comprehensive patient prognostic information to assess the clinical predictive value of *TRIM26*. Finally, although this study observed correlations between *TRIM26* expression and multiple immune cell infiltration levels, we did not probe into the specific molecular mechanisms. Therefore, future studies are encouraged to systematically evaluate the impact of TRIM26 on immune cell functional states and antitumor immune responses using flow cytometry, immunohistochemistry, and in vivo tumor models.

## 5. Conclusion

Overall, this study systematically described the expression pattern and clinical significance of *TRIM26* in HCC. According to integrated transcriptomic analyses, *TRIM26* was significantly upregulated in HCC tissues and exhibited a positive correlation with Wnt/*β*‐catenin pathway activity. Single‐cell profiling data further localized *TRIM26* expression primarily to hepatocytes, T/NK cells, myeloid cells, and B cells. Hepatocyte‐specific expression was positively associated with the Wnt pathway activity. These findings provided new insights into the cell type–specific role of *TRIM26* in HCC and highlighted its potential as a molecular link between Wnt signaling and the tumor immune microenvironment. From a translational perspective, *TRIM26* may serve as a potential prognostic biomarker, predictor of immunotherapy response, and therapeutic target candidate. Future functional studies are warranted to validate its mechanistic role and clinical applicability.

**Nomenclature**
APCs: Antigen‐presenting cells
API: Application programming interface
BRCA: Breast invasive carcinoma
CESC: Cervical squamous cell carcinoma
CHOL: Cholangiocarcinoma
ESCA: Esophageal carcinoma
FDR: False discovery rate
FC: Fold change
GDC: Genomic Data Commons
GEO: Gene Expression Omnibus
GSEA: Gene set enrichment analysis
HCC: Hepatocellular carcinoma
HBV: Hepatitis B virus
HCV: Hepatitis C virus
LIHC: Liver hepatocellular carcinoma
LUSC: Lung squamous cell carcinoma
LUAD: Lung adenocarcinoma
MSigDB: Molecular Signatures Database
MASH: Metabolic dysfunction‐associated steatohepatitis
MSI: Microsatellite instability
NKT: Natural killer T
ssGSEA: Single‐sample Gene Set Enrichment Analysis
scRNA‐seq: Single‐cell RNA sequencing
STAD: Stomach adenocarcinoma
TCGA: The Cancer Genome Atlas
TCRs: T cell receptors
TMB: Tumor mutation burden
UCEC: Uterine corpus endometrial carcinoma
ZEB1: Zinc finger E‐box‐binding homeobox 1

## Author Contributions

Conghua Song and Xiaomei Li designed this study. Zijun Hou acquired and interpreted the data. Huifeng Wu and Conghua Song drafted and revised the manuscript. Conghua Song and Zijun Hou contributed to this study equally.

## Funding

This study was supported by the Collaborative Project Between the Hospital and College of Putian University (2024106); the Joint Fund Project for Scientific and Technological Innovation in the Field of Medical and Health Care in Putian (2024SJYL079); the Fujian Provincial Health Technology Project (2024GGA089); the National Natural Science Foundation of China (82200676); and the Science and Technology Planning Project of Putian City (2023SZ3001PTXY01 and 2025NJYL069).

## Disclosure

All authors read and approved the manuscript.

## Ethics Statement

Ethical approval was not required for this study because it is not involved in any human or animal experiments.

## Consent

The authors have nothing to report.

## Conflicts of Interest

The authors declare no conflicts of interest.

## Supporting information


**Supporting Information** Additional supporting information can be found online in the Supporting Information section. Figure S1: Quality control metrics across cell clusters. (A) Violin plot of the number of genes detected per cell (nFeature_RNA); (B) violin plot of total UMI counts per cell (nCount_RNA) grouped by cluster; (C) violin plot of the percentage of mitochondrial gene expression relative to all genes per cell; and (D) UMAP visualization of the cell subpopulations.

## Data Availability

The datasets generated and/or analyzed during the current study are available in the [GSE149614] repository, [https://www.ncbi.nlm.nih.gov/geo/query/acc.cgi?acc=GSE149614].
