# Supplementary figures and images for: Transcriptomic Analysis Reveals the Role of TRIM26 in Hepatocellular Carcinoma and Its Association With the Wnt/β‐catenin Signaling Pathway
Source: Hum Mutat. 2026 Apr 24;2026:3090777. doi: 10.1155/humu/3090777 (PMC13108243; doi:10.1155/humu/3090777)

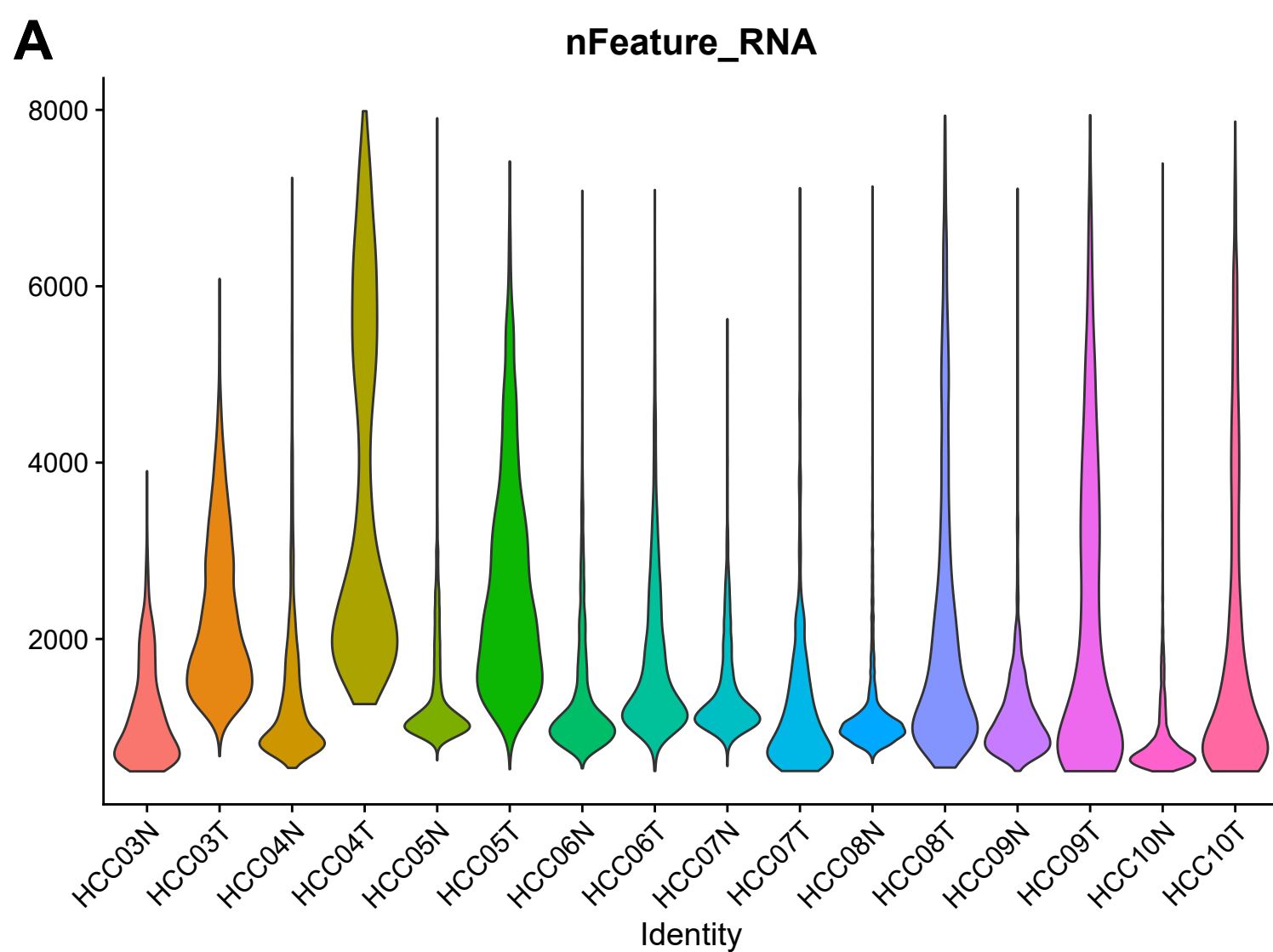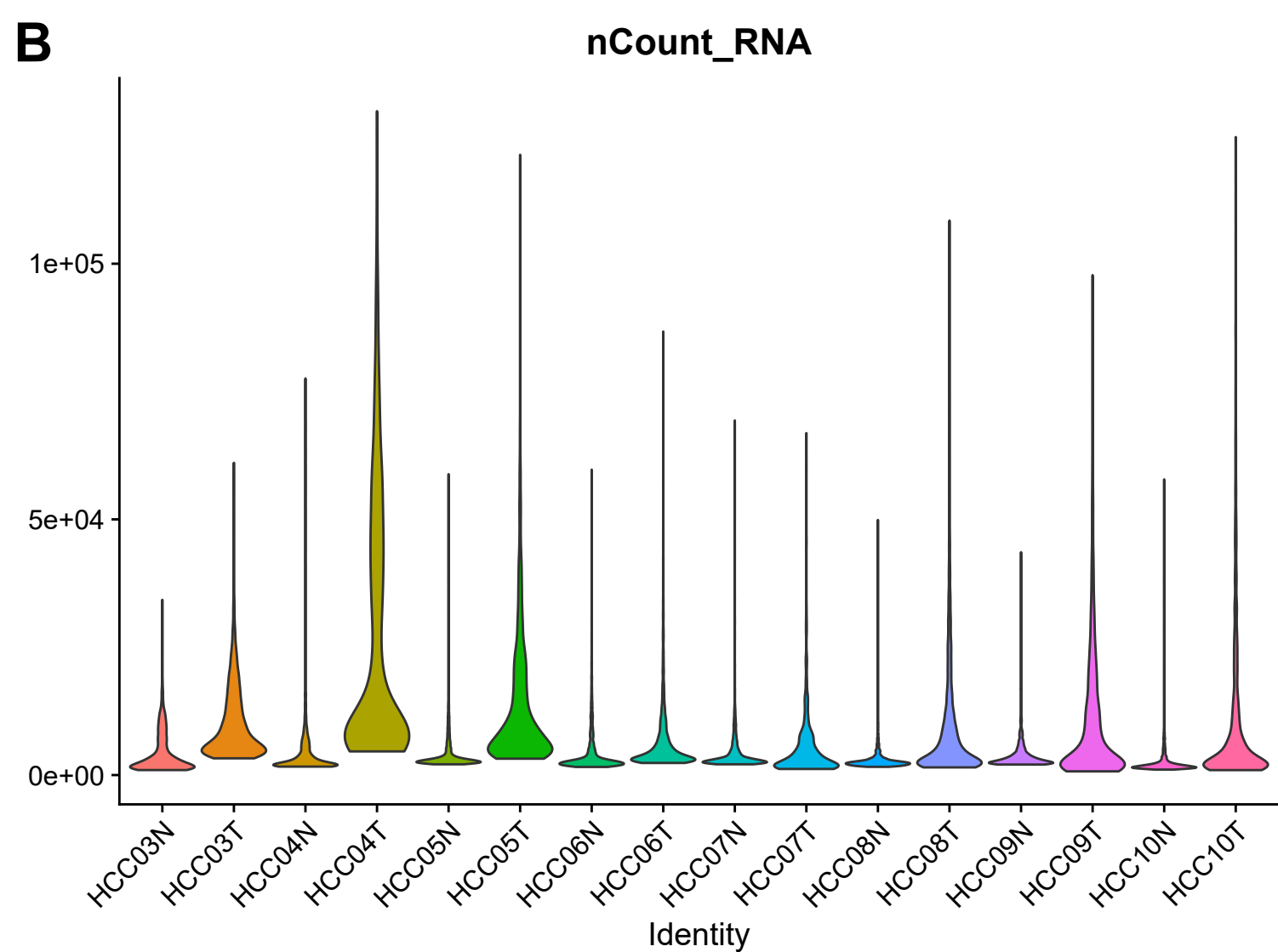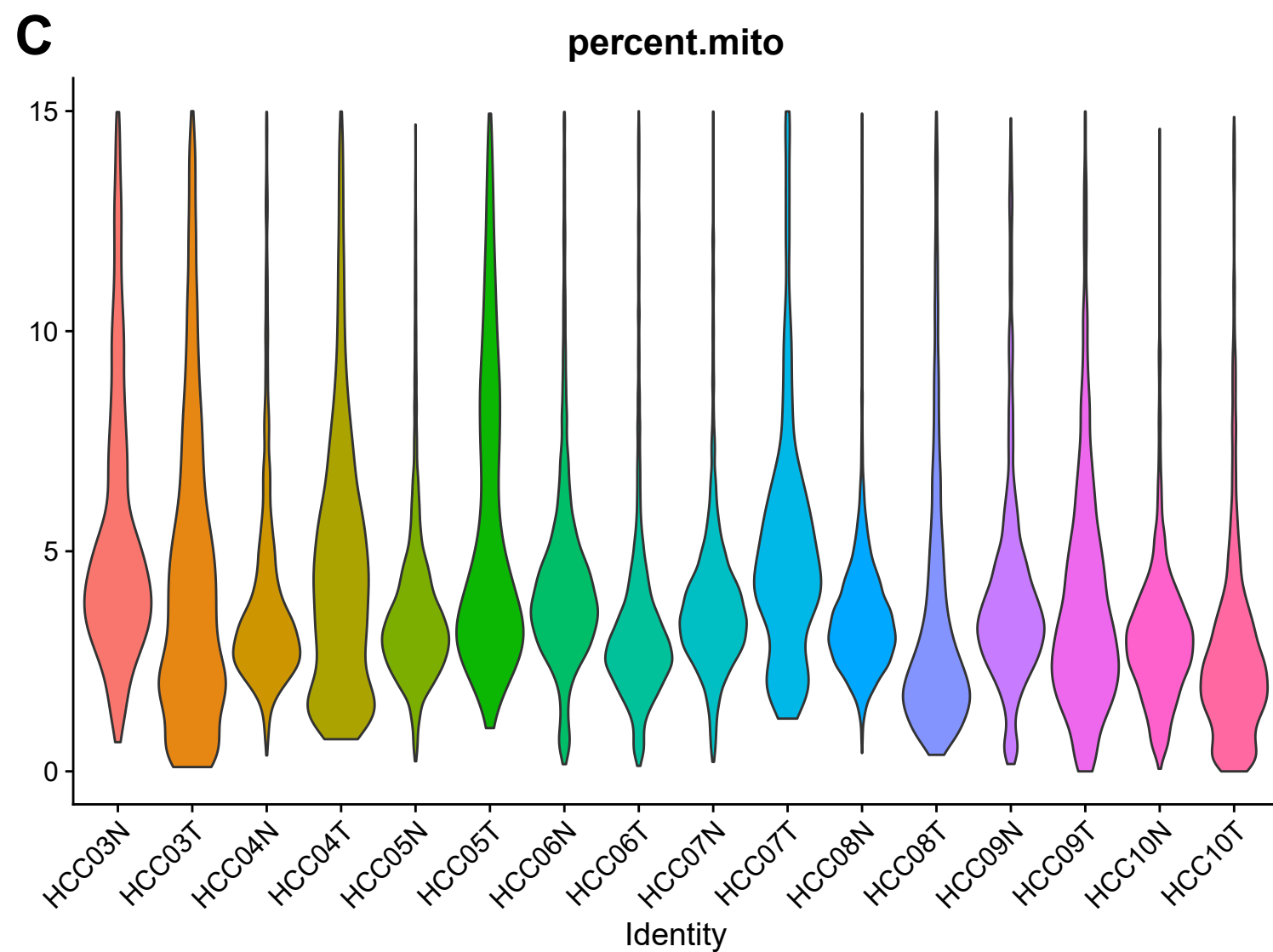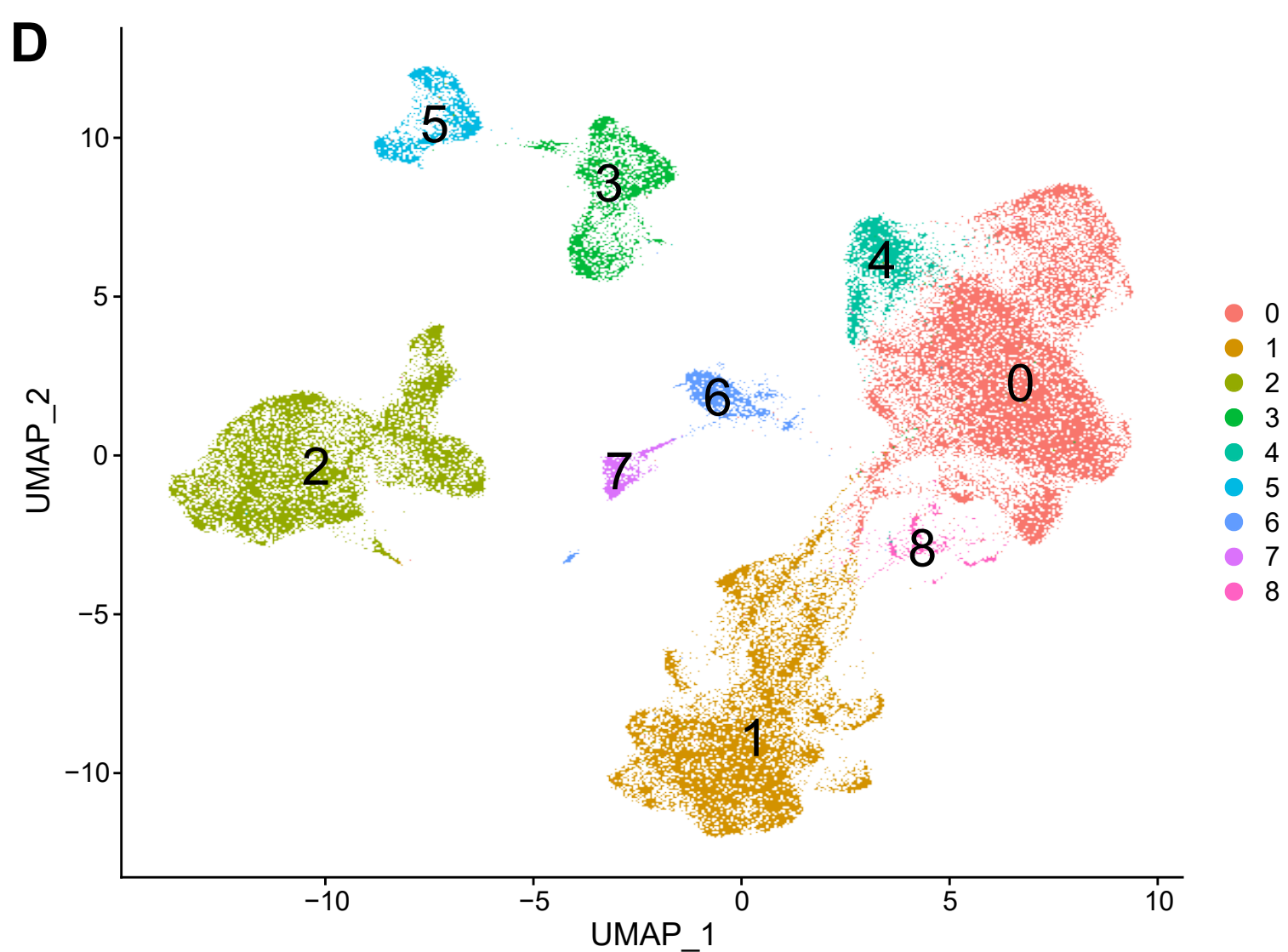

Supplement: Supplementary file 1 — Supporting Information Additional supporting information can be found online in the Supporting Information section. Figure S1: Quality control metrics across cell clusters. (A) Violin plot of the number of genes detected per cell (nFeature_RNA); (B) violin plot of total UMI counts per cell (nCount_RNA) grouped by cluster; (C) violin plot of the percentage of mitochondrial gene expression relative to all genes per cell; and (D) UMAP visualization of the cell subpopulations. [file HUMU-2026-3090777-s001.zip › Figure S1.pdf]
